# Patterns of primary care and mortality among patients with schizophrenia or diabetes: a cluster analysis approach to the retrospective study of healthcare utilization

**DOI:** 10.1186/1472-6963-9-127

**Published:** 2009-07-26

**Authors:** Laurel A Copeland, John E Zeber, Chen-Pin Wang, Michael L Parchman, Valerie A Lawrence, Marcia Valenstein, Alexander L Miller

**Affiliations:** 1 VERDICT Research, South Texas Veterans Health Care System, San Antonio TX, USA; 2 Department of Psychiatry, University of Texas Health Science Center at San Antonio, San Antonio TX, USA; 3 Department of Epidemiology and Biostatistics, University of Texas Health Science Center at San Antonio, San Antonio TX, USA; 4 Department of Family & Community Medicine, University of Texas Health Science Center at San Antonio, San Antonio TX, USA; 5 Department of Medicine, University of Texas Health Science Center at San Antonio, San Antonio TX, USA; 6 SMITREC Research, Veterans Administration Ann Arbor Healthcare System, Ann Arbor MI, USA; 7 Department of Psychiatry, University of Michigan, Ann Arbor MI, USA

## Abstract

**Background:**

Patients with schizophrenia have difficulty managing their medical healthcare needs, possibly resulting in delayed treatment and poor outcomes. We analyzed whether patients reduced primary care use over time, differentially by diagnosis with schizophrenia, diabetes, or both schizophrenia and diabetes. We also assessed whether such patterns of primary care use were a significant predictor of mortality over a 4-year period.

**Methods:**

The Veterans Healthcare Administration (VA) is the largest integrated healthcare system in the United States. Administrative extracts of the VA's all-electronic medical records were studied. Patients over age 50 and diagnosed with schizophrenia in 2002 were age-matched 1:4 to diabetes patients. All patients were followed through 2005. Cluster analysis explored trajectories of primary care use. Proportional hazards regression modelled the impact of these primary care utilization trajectories on survival, controlling for demographic and clinical covariates.

**Results:**

Patients comprised three diagnostic groups: diabetes only (n = 188,332), schizophrenia only (n = 40,109), and schizophrenia with diabetes (Scz-DM, n = 13,025). Cluster analysis revealed four distinct trajectories of primary care use: consistent over time, increasing over time, high and decreasing, low and decreasing. Patients with schizophrenia only were likely to have low-decreasing use (73% schizophrenia-only vs 54% Scz-DM vs 52% diabetes). Increasing use was least common among schizophrenia patients (4% vs 8% Scz-DM vs 7% diabetes) and was associated with improved survival. Low-decreasing primary care, compared to consistent use, was associated with shorter survival controlling for demographics and case-mix. The observational study was limited by reliance on administrative data.

**Conclusion:**

Regular primary care and high levels of primary care were associated with better survival for patients with chronic illness, whether psychiatric or medical. For schizophrenia patients, with or without comorbid diabetes, primary care offers a survival benefit, suggesting that innovations in treatment retention targeting at-risk groups can offer significant promise of improving outcomes.

## Background

Excess mortality has been documented among patients with schizophrenia, [1] and indeed schizophrenia has been estimated in community-based studies to be associated with up to 25 years' shorter lifespan. [2] Mortality rates for all causes, natural causes, and unnatural causes are all higher than expected among schizophrenia patients relative to the general population. [3]

Medical comorbidity among aging schizophrenia patients is common, the result of poor health behaviors, medication side effects, and schizophrenia itself, in addition to the usual functional and health status declines associated with aging. [4-7] Diabetes, in particular, represents a significant medical illness among individuals with psychiatric conditions. This comorbidity affects about 20% of all VA patients with or without a serious mental illness. In 2002, nearly 670,000 veterans over age 50 received care for diabetes in the VA, including 13,000 with schizophrenia.

The benefits of primary care for patients in general and for patients with a complex chronic illness, specifically, have been well documented. Multiple studies demonstrate a relationship between availability of primary care and mortality. [8,9] In small geographic areas where access to primary care is better, people are more likely to report improved overall health. [10] Delivery of high quality primary care is associated with a reduction in racial/ethnic health disparities for both physical and mental health. [8] Studies in both the U.S. and other countries consistently find that population health is better where there are more primary care providers. [9] Among patients with type 2 diabetes, higher levels of continuity with a primary care provider are associated with improved glucose control. [10] Furthermore, when primary care delivery is more consistent with the chronic care model, patients with diabetes have a reduction in likelihood of developing coronary heart disease. [11]

Although the delivery of primary care has documented benefit, health services use by patients with schizophrenia is frequently suboptimal. In the VA, veterans with schizophrenia are almost 40% less likely to have visited a primary care provider compared to patients without a psychiatric diagnosis over a one-year period. [12] Moreover, patients with schizophrenia are less likely to remain engaged in appropriate health care, although when mentally ill patients are "well-engaged" in care, appropriate care is more likely. [13-15] Dixon's research group noted diminished quality of care for patients with both serious mental illness (schizophrenia or major mood disorder) and diabetes, relative to patients with diabetes alone, in their study of quality indicators. [16] This is a troubling finding in view of the high level of risk factors for diabetes among VA patients with serious mental illness. Among inpatient decedents, loss of contact with the VA healthcare system for the year-long prior period was associated with increased risk of unforeseen death and was more common among patients with schizophrenia (20%) than among other decedents (8%). [17] If loss of system contact predicts subsequent increased risk of unforeseen death among inpatients, does decreased primary care put outpatients at increased risk? Seminal risk factors for mortality such as primary care utilization among the population of VA outpatients with schizophrenia should be identified to permit outreach to those patients at high risk of death.

The purpose of this study was to examine the relationships between patterns of primary care and mortality across three groups of patients: those with schizophrenia only, diabetes only, or both conditions.

## Methods

### Study Design

The local institutional review board approved the study prior to its commencement. Patient data for 241,508 VA patients with schizophrenia and/or diabetes were extracted from administrative sources (fiscal years 2002 – 2005) to provide measures of patient characteristics and care in the VA for this retrospective cohort study.

### Sample

Patients included in the study were aged 50 or older on Oct. 1, 2001 (fiscal year 2002 runs from October 2001 through September 2002) and carried a diagnosis of schizophrenia, diabetes mellitus, or both schizophrenia and diabetes (Scz-DM). Patients were considered to have schizophrenia if they had an ICD-9 code of 295.xx (excluding latent schizophrenia 295.5x) from at least one VA inpatient stay or at least two VA outpatient visits on different dates, based on the methods of the VA's National Psychosis Registry. [18] This definition selects only patients in contact with the healthcare system at baseline, and thus may not fully include intermittent users.

Patients with schizophrenia typically have other psychiatric conditions in the medical record. Patients with schizophrenia who were also diagnosed with schizoaffective disorder or other serious mental illness, including bipolar disorder, major depressive disorder, other psychosis, and post-traumatic stress disorder (ICD-9 codes 295.7; 296.0, 296.1, 296.4–296.8; 296.2–296.3; 297–298; 309.81) but were treated primarily for schizophrenia, as described above, were retained in the sample. Patients diagnosed more frequently with a serious mental illness other than schizophrenia or schizoaffective disorder (i.e., bipolar disorder, major depressive disorder, other psychosis, or post-traumatic stress disorder) were excluded from the study. [18]

Patients were identified as having diabetes if they had an ICD-9 code of 250.xx, 357.2x, 362.0x, or 366.41 on at least two different outpatient care dates in fiscal 2002 at least 30 days apart, consistent with diagnostic validity research. [19] Diabetes-only patients were limited to those without diagnosed serious mental illness, as defined above. Because of the very large number of diabetes patients, patients diagnosed with diabetes and no serious mental illness were randomly sampled and age-matched 1 to 4 with patients with schizophrenia.

### Data Sources

This study used archival data extracted from the VA's all-electronic medical record detailing patients' use of VA hospitals, extended care facilities, and outpatient clinics, as well as prescription medications, laboratory tests, and enrolment status. In the VA vital status database, date of death is based on VA, Social Security Administration, and US National Death Index records per a validated algorithm with demonstrated sensitivity of 98%. [20] The VA vital status database does not report cause of death.

### Measures

Race, gender, and marital status were recoded from medical record extracts as indicators of Hispanic, black, and missing race data, female gender, and married vs divorced vs widowed vs never married at baseline. Age was calculated as of the beginning of the study period, October 1, 2001. Date of death was used to calculate survival from the beginning of the study period.

The outpatient healthcare parameter of interest, primary care, was assessed by totalling the number of days each year on which patients had primary care visits (includes geriatric primary care and women's clinic). The trajectory of primary care use was captured by a cluster analysis of the number of primary care visits per year over the four years of the study. There are several advantages to this approach compared to total counts of visits. First, this approach permits inspection of differential use of primary care within years as well as across years, in contrast with the standard approach of analyzing across years only. In addition, this allows one to group patients according to their trajectory of primary care use, rather than by total utilization. The method is further described in the Analysis section.

Loss of system contact was determined from enrolment files for each follow-up fiscal year and summarized in an indicator (yes/no) denoting loss of contact during FY03–FY05, where patients scoring 0 or "no" were seen in the VA at least once during each year, and those scoring 1 were alive but had no VA care during at least one follow-up year.

In addition to the index diagnosis, measures of other illness were derived from ICD-9 codes on inpatient and outpatient records, including the Selim comorbidity indices. The Selim physical comorbidity index counts 30 medical conditions, although we excluded diabetes which was captured separately by diagnosis group. The indices were developed with self-report data, and have been operationalized and validated in VA administrative data. [21,22] We also counted the number of drug classes for which patients had VA prescriptions during each year, as this has been found to be a good proxy for comorbidity burden. [23] Interestingly, this measure was not highly collinear with the Selim index (variance accounted for = 28%).

Veteran priority score is assigned by the VA to veterans to determine eligibility for VA healthcare. It is associated with physical and mental health status, and it appears to be a proxy for socioeconomic status and disease severity. [24] It incorporates service-connected disability, a rating of how related a veteran's illness is to his/her military service, described in increments of 10% from 0% to 100%. The categories of VA priority status are: service-connected disability of 50% or more (category 1), service-connected disability 30% or 40% (category 2), former POWs, Purple Heart recipients, 10% or 20% disability (category 3), catastrophically disabled (category 4), very low income (category 5), special era-related status/0% service-connected disability (category 6), and non-service-connected status (categories 7, 8). [22,25] Priority 1 patients have no co-pays for VA care or prescriptions. At the time of this study, Priority 2–6 patients had co-pays for some VA care and for VA prescriptions; Priority 7–8 had co-pays for care and prescriptions.

### Analysis

Descriptive frequencies and means were prepared. We used cluster analysis to identify patterns of health services utilization, logistic regression to assess the relationship between patterns of care and death, and Cox proportional hazards to analyze associations with survival in the study period.

The cluster analysis explored the possibility of different trajectories of health care use within the sample. Cluster analysis classifies respondents by the pattern or profile defined by their values on specific variables (e.g., primary care visits each year). The profiles may differ in shape or scale. A difference in shape might indicate consistent values over time (a flat line) or might indicate successively increasing values over time (a sloped line). A difference in scale might occur when one group has consistently high scores while another has consistently low scores. In this type of cluster analysis, the distance between cases (persons) is calculated based on their values on the variables of interest (number of primary care visits during each of four years), and cases that are closest together in Euclidean space, i.e., that have the most similar pattern of values, are clustered together. The cluster analysis begins by clustering the closest pair, then forms the next cluster treating the first pair as a single unit, and so on until the number of clusters reduces from the number of cases (241,466) to the number of clusters with Eigenvalues greater than 1 (in this case, 4).

We did not impose a specific structure on the shapes of the trajectories over time. Thus for each cluster, the mean slope of the trajectory each year was the difference of the mean number of primary care visits between adjacent years, which could vary across years. To handle the large dataset, we executed a preliminary clustering to produce 20 clusters (PROC FASTCLUS, which optimizes processing by not assessing Eigenvalues) followed by a standard cluster analysis of the preliminary clusters, allowing the data to dictate the number of clusters in the final analysis (those with Eigenvalues greater than 1). We then inspected the proximity coefficients and the theoretical meaningfulness of the solutions. Ward's method, which uses squared Euclidean distances as a proximity measure, identified the cluster solutions. This method maximizes within-group homogeneity and between-group heterogeneity, and does not require pre-specification of the number of clusters. [26,27]

Logistic regression models assessed the relationship between death and patterns of care, adjusting for covariates (listed below). Logistic regression model fit was assessed by the concordance c-statistic, which ranges from 0.50 (no improvement over null) and 1.0 indicating perfect fit. Hazard ratios were estimated for primary care cluster by a Cox proportional hazards model, adjusting for covariates. Survival analysis modelled years survived from the beginning of the study period as a function of patterns of care (primary care cluster). Predictors included diagnostic group (schizophrenia, Scz-DM, diabetes), age as of October 1 2001, race, gender, marital status, loss of system contact, VA priority status, Selim physical comorbidity score, and number of medication classes prescribed in the baseline year. An interaction term of cluster by diagnosis was included to determine whether use of primary care had a differential impact on mortality over the 4-year study, for patients with schizophrenia, diabetes or both conditions.

## Results

### Sample

A total of 242,898 VA patients met eligibility criteria. Patients without valid priority status were considered to be non-veterans and were excluded from the study (n = 1,390); another 42 cases were dropped for utilization more than 31 days after date of death (post-mortem bereavement counselling is offered by the VA; continued illogical use suggests error). The final sample numbered 241,466 VA patients

There were 53,134 schizophrenia patients aged ≥ 50 years with valid priority status receiving care in the VA in FY02. These patients were age-matched in a 4:1 ratio with diabetes patients (n = 188,332). During matching, it was noted that there were not enough diabetes patients aged 50–52 to match 4:1 for all schizophrenia patients; this age group matched 2.5 diabetes patients per 1 schizophrenia patient. Among patients with schizophrenia, 25% (n = 13,025) were also diagnosed with diabetes leaving 40,109 patients in the schizophrenia-only group.

The sample included 2.2% female veterans and ranged in age from 50 to 104 years (mean = 60.7; SD = 9.5). Twenty-one percent (21%) of patients were of non-white race/ethnicity (African-American, Hispanic), 53% were white, and 26% had missing data on this measure. Over the 4 years of the study, 5.5% of patients were not seen in the VA during one or more fiscal years in which they were alive; 14.5% of the patients died (see Table 1).

**Table 1:** Sample Characteristics of VA Patients with Diabetes, Schizophrenia, or Both Diabetes and Schizophrenia (N = 241,466)

**Characteristic**	**Schizophrenia** **Only** **(n = 40,109)**	**Both** **Schizophrenia** **& Diabetes** **(n = 13,025)**	**Diabetes Only** **(n = 188,332)**	**Overall Sample** **(N = 241,466)**
	**Mean (SD)**			

Age (range 50–104 years)	59.8 (9.5)	60.4 (9.3)	61.0 (9.5)	60.7 (9.5)

Age at Death (n = 34,991)	68.1 (10.9)	67.7 (10.4)	68.6 (11.0)	68.5 (10.9)

	**N (%)**			

Female	1,359 (3.4)	439 (3.4)	3,429 (1.8)	5,227 (2.2)

Race/Ethnicity				

Hispanic	2,786 (7.0)	1,411 (10.8)	10,440 (5.5)	14,637 (6.1)

Black	8,482 (21.2)	3,532 (27.1)	23,745 (12.6)	35,759 (14.8)

White	24,195 (60.3)	6,920 (53.1)	96,084 (51.0)	127,199 (52.7)

Other/unknown	4,286 (10.7)	1,003 (7.7)	56,451 (30.0)	61,740 (25.6)

Marital Status				

Married	9,806 (24.5)	4,286 (32.9)	117,870 (62.6)	131,962 (55.3)

Divorced	11,462 (28.6)	3,608 (27.7)	39,351 (20.9)	54,421 (22.8)

Never married	16,481 (41.1)	4,338 (33.3)	19,536 (10.4)	40,355 (16.9)

Widowed	1,683 (4.2)	657 (5.0)	9,751 (5.2)	12,091 (5.1)

Diagnosis Group	40,223 (16.6)	13,057 (5.4)	189,618 (78.1)	--

Diagnosis of Hypertension	14,187 (35.4)	8,252 (63.4)	139,214 (73.9)	161,653 (67.0)

Diagnosis of Hyperlipidemia	9,300 (23.2)	5,590 (42.9)	103,965 (55.2)	118,855 (49.2)

Diagnosis of Dementia	1,123 (2.8)	414 (3.2)	1,142 (0.6)	2,679 (1.1)

Any Inpatient Admission over study period	21,550 (53.7)	8,137 (62.5)	63,317 (33.6)	93,004 (38.5)

Priority Category				

Category 1: 50–100% disabled	20,705 (51.6)	7,440 (57.1)	55,106 (29.3)	83,251 (34.5)

Category 2: 30–40% disabled	1,226 (3.1)	467 (3.6)	13,268 (7.1)	14,961 (6.2)

Category 3: 10–20% disabled, former POW, Purple Heart	1,694 (4.2)	707 (5.4)	18,086 (9.6)	20,487 (8.5)

Category 4: Catastrophically disabled	8,188 (20.4)	2,248 (17.3)	12,905 (6.9)	23,341 (9.7)

Category 5: Low income	7,748 (19.3)	2,037 (15.6)	64,662 (34.3)	74,447 (30.8)

Category 6: 0% disabled, special eras	--	--	--	781 (0.3)

Category 7–8: Non-service-connected status (copayment required)	504 (0.2)	117 (0.9)	23,577 (12.5)	24,198 (10.0)

**Figure 1:**
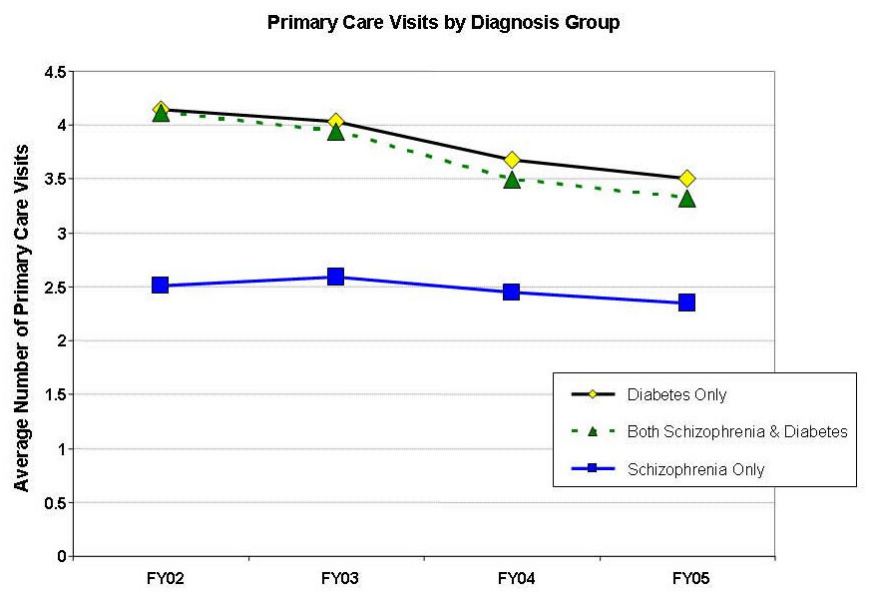
**Average Primary Care Use by VA Patients with Schizophrenia, Diabetes, or Both Conditions over 4 Years (N = 241,508)**.

Characteristics varied by diagnostic group. Patients with diabetes only were more likely to be married (63%) or widowed (5.2%) compared to schizophrenia patients, whereas patients with schizophrenia, regardless of diabetes status, were more likely to have never married (diabetes 10% vs Scz-DM 34% vs schizophrenia 42%; chi-square(6) = 32,140; p < .0001). Patients diagnosed with schizophrenia were more likely to be female (schizophrenia: 3.4% female, Scz-DM: 3.4% female, diabetes: 1.8% female; chi-square(2) = 478; p < .0001). Diabetes patients had the lowest rates of death (13.7%; chi-square(2) = 589; p < .0001), compared to schizophrenia patients (16.3%) and Scz-DM patients (20.6%). Average primary care use (mean, SD) is shown in Figure 1.

Missing data on race was three times more common among diabetes patients relative to schizophrenia patients (diabetes 30% vs Scz-DM 8% vs schizophrenia 11% missing race data; chi-square(2) = 8774; p < .0001). In VA databases in 2002, race was recorded by a clinical observer in inpatient records and was rarely missing, while outpatient records were characterized by high rates of missing data. Because patients with schizophrenia are likely to be hospitalized for acute psychotic exacerbations, they had more opportunity to receive a valid code on this measure.

### Comorbidity

Among patients diagnosed with schizophrenia, 34% were also diagnosed with schizoaffective disorder, post-traumatic stress disorder (7%), bipolar disorder (4%), major depression (3%) or other psychosis (3%) on two or more occasions in the baseline year.

The Selim physical comorbidity score, minus diabetes, averaged 2.3 (SD = 1.6; range 0–15) for patients with diabetes alone, 2.1 (SD = 1.6; range 0–11) for patients with both index conditions, but 1.4 (SD = 1.5; range 0–13) for patients with schizophrenia alone. The average number of medication classes for which patients received prescriptions in the baseline year was 6.4 (SD = 4.5; range 0–33) for schizophrenia-only patients, 10.0 (SD = 5.3; range 0–36) for both conditions, 8.7 (SD = 4.7; range 0–42) for diabetes-only.

### Cluster Profiles

**Figure 2:**
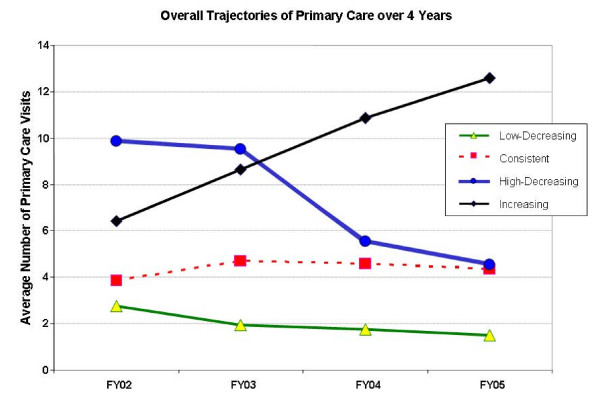
**Primary Care Trajectories over the period FY02–FY05 for VA Patients with Schizophrenia, Diabetes, or Both Conditions (N = 241,508)**.

The preliminary cluster analysis produced 20 clusters with between-centroid distances ranging from 3.6 to 345.5. The final cluster analysis identified four distinct patterns of primary care visits over the 4-year study period (shown in figure 2). The first cluster depicted Increasing use of primary care from an average of 6 to 13 visits per year. The second cluster contained patients whose trajectory of primary care use was Consistent, averaging approximately 4 visits per year. Cluster three profiled a trajectory of decreasing use of primary care at a low level (Low-decreasing), falling from about 3 visits per year to about 1.5 visits per year. The fourth and final cluster described a trajectory of high levels of primary care use decreasing sharply from 10 to 4.5 visits per year on average (High-decreasing).

**Figure 3:**
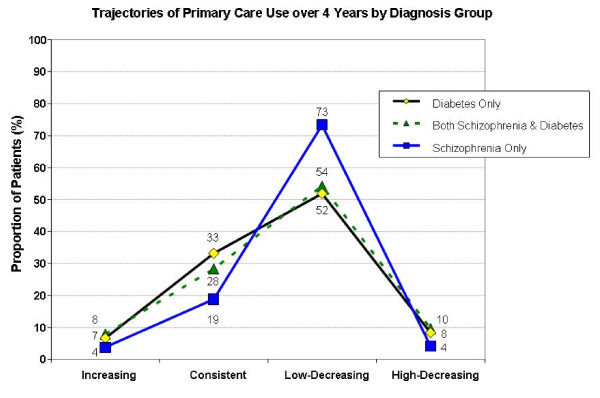
**Trajectories of Primary Care Use by Older VA Patients with Chronic Disease: Schizophrenia Only, Diabetes Only, or Both Schizophrenia & Diabetes**.

While most patients were in the low-decreasing primary care cluster, cluster membership differed by diagnostic group, as illustrated in Figure 3. Patients with schizophrenia only were predominant in the Low-decreasing trajectory, those with diabetes-only dominated the Consistent use trajectory, and Scz-DM patients were the most numerous group in both the Increasing and High-decreasing trajectories. Consistent-use cluster membership was more common among diabetes patients (33%) than among Scz-DM (28%) or schizophrenia-only patients (19%). Patients with schizophrenia were more likely to experience the low-decreasing trajectory of primary care, 73% schizophrenia-only vs 54% Scz-DM vs 52% diabetes-only. High-decreasing primary care use was associated with diabetes, with or without schizophrenia: 10% of Scz-DM patients experienced this trajectory, 8% of diabetes patients, and 4% of schizophrenia-only patients.

### Mortality Models

An unadjusted model of mortality during the three years of follow-up estimated increased relative odds of death associated with membership in Low-decreasing primary care use compared to the Consistent-use cluster (OR = 3.8, 95% CI 3.7–4.0). Compared to patients with Consistent primary care use, patients in the Increasing-use trajectory had lower relative odds of death (OR = 0.50, CI95 .45–.55) while those with High-decreasing use had increased odds of death (OR = 2.5, CI95 2.3–2.6). The unadjusted model had a poor fit as assessed by the C-statistic of 0.64.

The adjusted model showed a significant interaction of diagnosis by primary care cluster (Wald chi-square(6) = 70.9; p < .0001) and a good fit (C-statistic = 0.78), with main effects of cluster and diagnosis. Additional factors associated with death during follow-up included loss of system contact (OR = 1.4), greater age (OR = 1.9 per decade), and increased comorbidity burden (OR = 1.1 per Selim physical comorbidity; 95% confidence intervals provided in Table 2). Being married (OR = 0.69) or female (OR = 0.64) was protective. The changes in estimated odds ratios from the unadjusted to the adjusted model demonstrate that some variation in mortality risk is attributable to specific aspects of health care, including treatment of multimorbidity and prescriptions, but significant variation in mortality is attributable to variation in the use of primary care. Relative odds of death associated with membership in each diagnosis by primary care sub-group, relative to a single reference group of diabetes with consistent care, are shown in the table.

**Table 2:** Characteristics Associated with Decreased Survival among VA Patients with Diabetes, Schizophrenia, or Both Diabetes and Schizophrenia (N = 241,466)

**Parameter**	**Hazard Ratio**	**Pr > ChiSq**
Age in Decades (range 5–10)	1.72	<.0001

Female	0.69	<.0001

Married	0.74	<.0001

Black	0.99	0.5030

Hispanic	1.00	0.9348

Missing Data on Race	0.73	<.0001

Vietnam Era	1.05	0.0023

Priority Status – Referent: Priority 1		

Priority Status 2	0.93	0.0046

Priority Status 3	0.96	0.0496

Priority Status 4	1.36	<.0001

Priority Status 5	1.01	0.5910

Priority Status 6	0.95	0.6357

Priority Status 7	0.79	<.0001

Priority Status 8	0.74	0.0582

Selim Physical Comorbidity Score (range 0–15)	1.10	<.0001

Number of Medication Classes at Baseline (range 0–42)	1.07	<.0001

Loss of System Contact During Study Period	1.17	<.0001

Primary Care Trajectory by Diagnosis Subgroups – Referent: Consistent Care – Diabetes Only		

Increasing Primary Care – Schizophrenia	0.26	<.0001

Consistent Primary Care – Schizophrenia	0.95	0.2781

Low-Decreasing Primary Care – Schizophrenia	4.20	<.0001

High-Decreasing Primary Care – Schizophrenia	1.31	0.0002

Increasing Primary Care – Schizophrenia with Diabetes	0.48	<.0001

Consistent Primary Care – Schizophrenia with Diabetes	1.01	0.8176

Low-Decreasing Primary Care – Schizophrenia with Diabetes	5.01	<.0001

High-Decreasing Primary Care – Schizophrenia with Diabetes	1.44	<.0001

Increasing Primary Care – Diabetes Only	0.38	<.0001

Low-Decreasing Primary Care – Diabetes Only	3.85	<.0001

High-Decreasing Primary Care – Diabetes Only	1.59	<.0001

**Figure 4:**
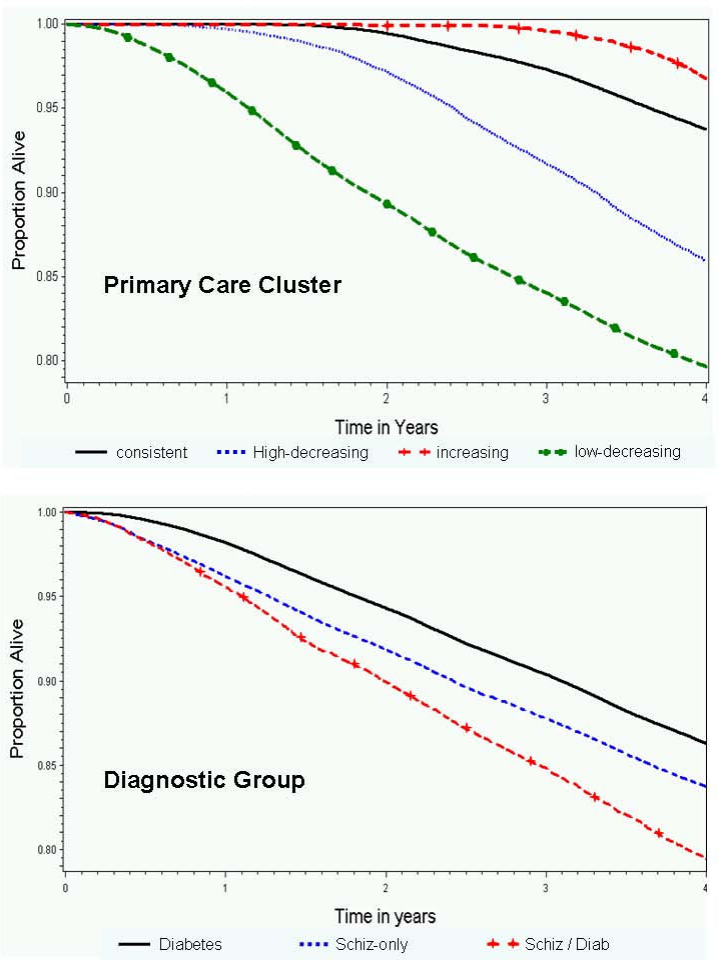
**Survival Curves by Primary Care Cluster, Diagnostic Group**.

In the covariate-adjusted Cox proportional hazards model of survival time, the interaction of primary care cluster by diagnosis was again significant. Hazard rates of mortality were similar within diagnosis groups for patients with either Increasing or High-decreasing primary care. The hazard ratio of mortality for schizophrenia and Scz-DM, relative to diabetes only, was significantly greater when the patient was experiencing low-decreasing primary care, compared to other trajectories of primary care. Additional risk of diminished survival was imparted by loss of system contact (HR = 1.2), older age (HR = 1.7 per decade), and more physical comorbidity (HR = 1.2 per point on the Selim score). Marriage (HR = 0.74) and female gender (HR = 0.69) were protective, as was Increasing primary care use. Compared to consistent use-diabetes patients, patients with increasing use were at much less risk of shorter survival (HR = 0.26 for schizophrenia patients, HR = 0.48 for Scz-DM patients, and HR = 0.38 for diabetes-only patients). Survival curves are shown in Figure 4.

## Discussion

Our cluster analysis approach to varying trajectories of primary care use allowed us to examine administrative data on a large sample of patients to distinguish important subgroups of patients for their differential risk of mortality. Survival was associated with patients' trajectory of primary care use, with differential effects by diagnosis group (schizophrenia only, diabetes only, both schizophrenia and diabetes). Overall, the interaction effect of diagnosis by primary care trajectory was primarily due to significantly greater hazard ratios in the low-decreasing cluster. Therefore, a pattern of primary care use that was either consistent over time or high-decreasing over time would be most likely to achieve optimal outcomes for patients with schizophrenia. Conversely, effective intervention with patients with low-decreasing primary care should have the best chance of reducing premature mortality, especially for patients with schizophrenia only as that group appears to be over-represented in the trajectory.

Why would decreasing use of primary care be associated with higher risk of mortality? Starfield and Shi, in their review of the benefits of primary care, postulate 6 mechanisms that may account for the beneficial effects of primary care: 1) increased access to health care services created by primary care for relatively deprived population groups, such as those with schizophrenia; 2) enhancement by primary care of overall quality of health care delivered to the patient; 3) impact of primary care on prevention; 4) impact of primary care on early management of health problems; 5) accumulated contribution of primary care characteristics such as care coordination to more appropriate care, for example, continuity of primary care and coordination of care are associated with better control of risk factors for cardiovascular disease such as blood pressure and lipid levels, especially for patients with diabetes; [11] and 6) the role of primary care in reducing unnecessary care that may lead to harm. [9] While schizophrenia-only patients in this study did not have diabetes, they frequently had comorbid hypertension or hyperlipidemia and were at risk from diabetogenic medications. [28]

Patients with decreasing primary care use may have substituted inpatient for outpatient care. In the VA, inpatients may still receive care in the outpatient clinics, because both types of care are provided in the larger facilities, making it difficult to disentangle these measures, but inpatient days did show a small negative correlation with primary care visits (r = -0.03 to r = -0.04). Thus, acutely ill patients requiring hospitalization could account for some of the shortened survival noted in this trajectory.

A limitation of this study is its reliance on VA administrative data; out-of-system health care use was not captured and qualitative data was not available. The scope of the study precluded chart review of the electronic medical records from which the administrative data were extracted. VA patients are predominantly male, reflecting enlistment practices of the armed services, but they also tend to be poorer and sicker than US residents in general, characteristics shared with low-income populations in general, such as Medicaid patients. Another limitation of the study is its bias against intermittent users of VA healthcare services, because inclusion in the cohort required at least two outpatient visits in the baseline year. Potentially a fifth trajectory of primary care exists in which intermittent users variably use primary care; our study is unable to address these possibilities. We know that a fairly large percent of VA patients, especially older individuals, utilize out of system providers (primarily through Medicare, the federal healthcare insurance program available to US residents over age 65), and this study was unable to examine such utilization. However, seriously mentally ill patients have been documented to remain within the VA for their care more often than other veterans. [29]

The relative value of psychiatric care vs primary care may be of interest, as these two forms of care may covary or may diverge, depending on the patient. We noted a modest positive correlation of psychiatric with primary care (r = .02 to r = .09). An in-depth exploration of this covariance may yield valuable insight.

Other protective factors for these patients were continued contact with the healthcare system, being married, being younger, and female gender. Loss of system contact was shown in previous research to be associated with unforeseen inpatient death and again in this study of outpatients. This study reinforces the value of at least annually using the VA healthcare system, so as to allow clinicians to review treatment recommendations, including adherence to psychotropic medications, monitoring blood glucose and blood pressure, and screening for new problems. Therefore, outreach to patients who have been out of contact for more than 12 months seems warranted, [30] in addition to maintaining consistent or higher levels of primary care.

## Conclusion

Older VA patients have a variety of risk factors for premature death, including comorbidity burden and sub-optimal use of health care services. Life might be extended by the timely treatment of comorbid physical disease. While research supports an inverse association between primary care and mortality, assessing patterns of primary care use in a cohort of patients offers the potential to illuminate which patients require outreach, and what patterns of care may be necessary to improve patient outcomes.

## Competing interests

The authors declare that they have no competing interests.

## Authors' contributions

LAC conceptualized the study, conducted the analyses, drafted and finalized the manuscript. JEZ contributed to the conceptual framework and writing. CPW provided statistical advice on modelling and interpretation and contributed to the writing of the manuscript. MLP advised on structuring the paper, wrote about primary care and diabetes management, and gave input to model design. VAL advised on late-life clinical issues, wrote about the role of primary care clinicians in inpatient care, and critiqued the manuscript. MV contributed to study development and the final manuscript. ALM participated in conceiving the study, interpreting study results, and finalizing the manuscript. All authors read and approved the final manuscript.

## Pre-publication history

The pre-publication history for this paper can be accessed here:


